# Characterization of monoclonal antibodies against waterfowl parvoviruses VP3 protein

**DOI:** 10.1186/1743-422X-9-288

**Published:** 2012-11-23

**Authors:** Xiuchen Yin, Shumei Zhang, Youlan Gao, Jinzhe Li, Shuyi Tan, Hongyu Liu, Xiaoying Wu, Yuhuan Chen, Ming Liu, Yun Zhang

**Affiliations:** 1 State Key Lab of State Key Laboratory of Veterinary Biotechnology, Harbin Veterinary Research Institute, CAAS, Harbin, 150001, China; 2 Hainan Key Lab of Tropical Animal Reproduction & Breeding and Epidemic Disease Research, Haikou, 571100, China

**Keywords:** Monoclonal Antibody, GPV, MDPV, VP3

## Abstract

**Background:**

The VP3 protein of goose parvovirus (GPV) or Muscovy duck parvovirus (MDPV), a major structural protein, can induce neutralizing antibodies in geese and ducks, but monoclonal antibodies (MAbs) against VP3 protein has never been characterized.

**Results:**

Three hybridoma cell lines secreting anti-GPV VP3 MAbs were obtained and designated 4A8, 4E2, and 2D5. Immunoglobulin subclass tests differentiated them as IgG2b (4A8 and 4E2) and IgG2a (2D5). Dot blotting assays showed that three MAbs reacted with His-VP3 protein in a conformation-independent manner. A competitive binding assay indicated that the MAbs delineated two epitopes, A and B of VP3. Immunofluorescence assay showed that MAbs 4A8, 4E2, and 2D5 could specifically bind to goose embryo fibroblast cells (GEF) or duck fibroblast cells (DEF) infected with GPV and MDPV. Dot blotting also showed that the MAbs recognized both nature GPV and MDPV antigen. Western blotting confirmed that the MAbs recognized VP3 proteins derived from purified GPV and MDPV particles. The MAbs 4A8 and 2D5 had universal reactivity to heterologous GPV and MDPV tested in an antigen-capture enzyme-linked immunosorbent assay.

**Conclusions:**

Preparation and characterization of these the MAbs suggests that they may be useful for the development of a MAb-capture ELISA for rapid detection of both GPV and MDPV. Virus isolation and PCR are reliable for detecting GPV and MDPV infection, but these procedures are laborious, time-consuming, and requiring instruments. These diagnosis problems highlight the ongoing demand for rapid, reproducible, and automatic methods for the sensitive detection of both GPV and MDPV infection.

## Background

Parvovirus infection is widespread in most goose farming countries of Europe and Asia and causes serious economic loss [1,2]. Goose parvovirus (GPV) can cause disease characterized by ascites, enteritis, myocarditis, and hepatitis with high mortality and morbidity in geese (Anser anser) and Muscovy ducks (Cairina moschata) [3-5].

The genomes of GPV and Muscovy duck parvovirus (MDPV) are 5106 nucleotides in length and contain two open reading frames (ORF). The left ORF encodes the regulatory proteins, whereas the right ORF encodes three capsid proteins: VP1, VP2, and VP3. VP1, VP2, and VP3 are derived from the same gene by differential splicing, and VP2 and VP3 are contained within the carboxyl-terminal portion of VP1 [6-8]. The VP1 polypeptides of GPV and MDPV share 88% amino acid sequence identity [7-9], which allows cross-protection of Muscovy ducks against MDPV infection by vaccination with attenuated GPV [10]. The VP3 protein is the most abundant of the three core proteins [11] and can induce neutralizing antibodies in GPV- or MDPV-infected waterfowls [12]. Recently, a new divergent MDPV (PSU-3101) has been isolated, which showed 84.5% sequence identity with other MDPV isolates and 84.6% identity with GPV isolates [13].

Many methods have been developed for the diagnosis of GPV or MDPV infections, including agar gel precipitation, the virus neutralization test [14], Western blotting assays [15], virus antigen-based enzyme-linked immunosorbent assays (ELISA) [10,16], the VP3 protein based ELISA [17], a plaque neutralization assay [18], an indirect fluorescent antibody test [19], polymerase chain reaction (PCR) for the rapid detection of GPV DNA [20], and quantitative analysis of waterfowl parvovirus by real-time PCR [21]. However, these methods are generally time-consuming and labor-intensive, and require sophisticated instruments. Here, we produced and characterized three MAbs against bacterially expressed VP3 protein of GPV. Contrary to currently available PCR tools, the MAbs could not discriminate between GPV and MDPV infection; however, due to 4A8 and 2D5 universal reactivity to both GPV and MDPV, the MAbs are ideal candidates for both GPV and MDPV clinical diagnosis in an antigen-capture ELISA.

## Results

### Production and characterization of MAbs

Three weeks after cell fusion, the hybridoma cell lines secreting anti-VP3 antibody were screened by means of an ELISA. Three MAbs directed against VP3 were selected and subcloned at least three times using the limiting dilution method. Hybridomas were selected to produce MAbs in mice and the ascitic fluids were used for further characterization. The isotypes of the MAbs were IgG2b (4A8 and 4E2) and IgG2a (2D5). Concentrations of immunoglobulin ranged from 0.40 to 17.53 μg/ml.

### Effect of denaturation of VP3 on MAb recognition

**Figure 1:**
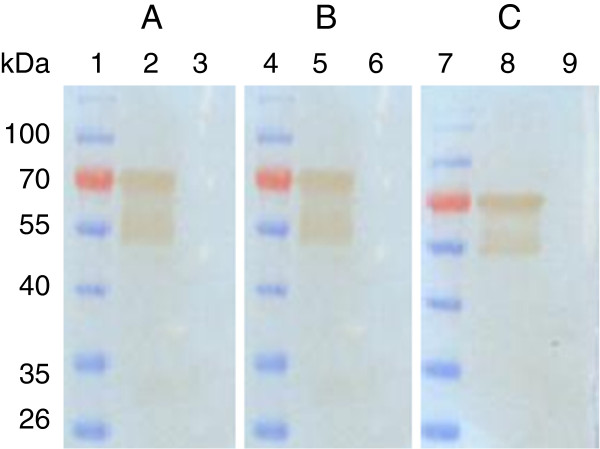
**Reactivity of Ep22 VP3 MAbs to the *E coli *expressed pET30-VP3 and pET30a vector.** Lane 1, 4, and 7, protein molecular marker; lane 2, 5, and 8 *E. coli* expressed pET30-VP3; lane 3, 6, and 9 *E.coli* expressed pET30a vector. MAb 4A8(**A**), MAb 4E2(**B**), and MAb 2D5(**C**).

**Figure 2:**
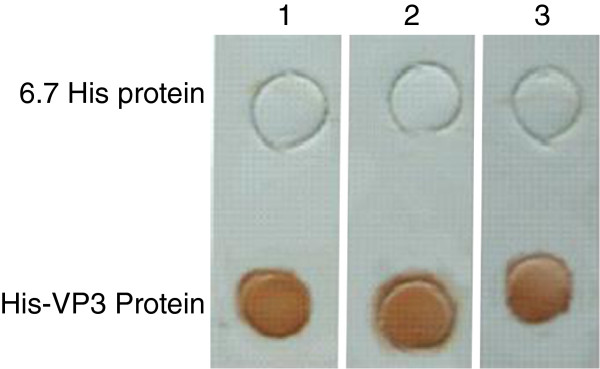
**Dot blotting assay of MAbs to the His-VP3 and His proteins.** Lane 1, MAb 4A8; lane 2, MAb 2D5; lane 3, MAb 4E2.

**Figure 3:**
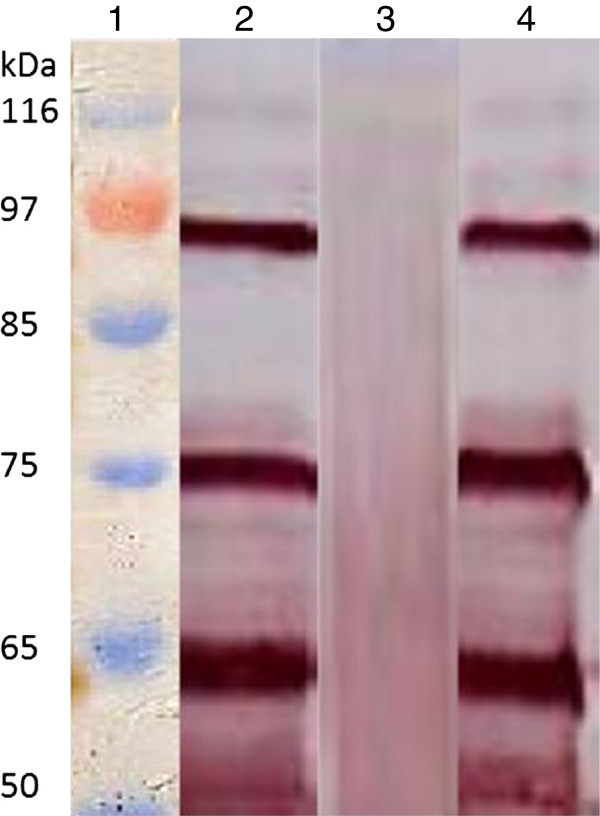
**Western blot analysis of purified GPV and MDPV to MAbs.** Lane 1: protein molecular weight marker; Lane 2: purified GPV EP22; Lane 3: blank allantoic fluids; Lane 4: purified MDPV J3D6.

The expressed His-VP3 proteins were denatured by boiling in SDS and 2-mercaptoethanol, and subjected to Western blotting; three MAbs still recognized them (Figure 1). To determine whether a VP3 structure is required for antibody binding, antigens containing His-VP3 were examined by using a dot blotting assay. All MAbs recognized the nature structure of His-VP3 in TNE buffer (Figure 2), but did not react with the 6.7 His proteins. Three major proteins (VP 1, VP 2 and VP 3) were identified by SDS-PAGE in a purified Muscovy duck (91, 78 and 58 kDa) and goose (85, 61 and 57 kDa) parvovirus virions [22-24]. Western blotting analysis showed that MAbs reacted with molecular weight of 90, 77, and 64 kDa of VP1, VP2, and VP3 from denatured GPV EP22 (represented by Figure 3, lane 2) and MDPV J3D6 (represented by Figure 3, lane 4) antigen, which were similar to VP1, VP2, and VP3 of goose parvovirus and Muscovy duck parvovirus. MAbs did not recognize blank allantoic fluids (represented by Figure 3, lane 3). This result suggested that the MAbs recognized VP3 proteins derived from purified GPV and MDPV particles. 

### Detection of native VP3 protein by immunofluorescence assay

**Figure 4:**
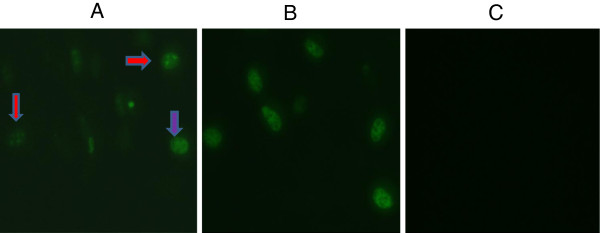
**Detection of VP3 protein by indirect immunofluorescence assay on cells infected with EP22 and J3D6.A**: GPV infected GEF, **B**: MDPV infected MDF, **C**: Mock infected cells. No special fluorescence was found on normal cells (400 ×).

Immunofluorescence assay was performed on EP22 and J3D6 infected GEF/DEF to assess whether the MAbs recognize the native-form of VP3 protein of GPV and DPV. Three MAbs strongly reacted with EP22 infected GEF cells or J3D6 infected DEF (represented by Figure 4A and B). All uninfected cells showed no reaction to any MAbs (represented by Figure 4C). Immunofluorescence assay also indicates that the MAbs bound to the authentic viral VP3 protein, which located predominantly within the nuclei without affecting the nucleoli and rarely within the cytoplasm of infected cells, which is consistent with previous report [22,23]. In some cells GPV/MDPV appeared as granules scattered throughout the nucleus (indicated by red arrow), while in other cells GPV/MDPV were distributed homogeneously in nuclei (indicated by purple arrow or in Figure 4B). 

### Effect of native structure of VP3 on MAbs recognition

**Figure 5:**
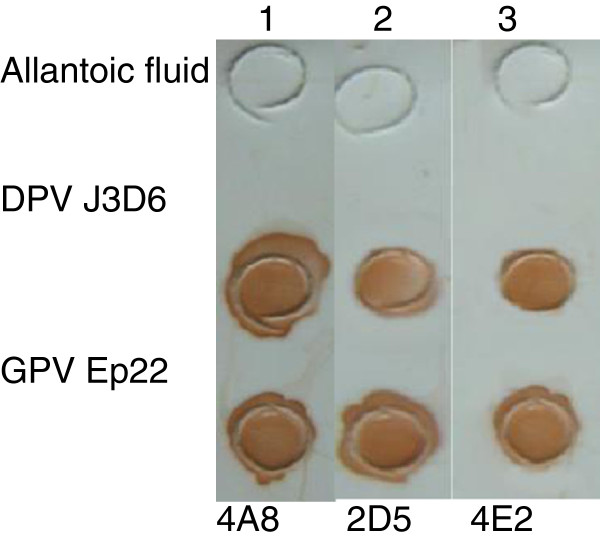
**Dot blotting assay of MAbs to native GPV and MDPV particles.** Lane 1, MAb 4A8, lane 2, MAb 2D5; lane 3, MAb 4E2.

Dot blotting assays showed that three MAbs recognized both GPV and DPV native particles, while blank allantoic fluids were not detected by any MAbs (Figure 5).

### Avidity of the MAbs to VP3

The amount of MAbs bound to the VP3 proteins can be quantified within the linear range of absorbance. This offers an estimation of the relative avidity of MAbs for their binding proteins. The binding degrees of the MAbs to the His-VP3, using ELISA titration, indicated that the three MAbs saturated fat dilutions ranging from 10^-1^ to 10^-1.6^. The three MAbs retained their binding capacity after coupling to HRP, and the dilution range of saturation was 10^1^ to 10^2^. No saturation was apparent with the remaining HRP-MAbs (data not shown).

### Epitopes mapping

The appropriate concentrations for the competitive binding assay were determined by using the dose–response curves plotted for the unconjugated and HRP-conjugated MAbs (data not shown). Each of the three MAbs was used both as a competitor and as an HRP-conjugated probe. The percentage of competition was 100% in the presence of a saturating unlabeled homologous antibody. Two distinct epitopes on VP3 were found and designated A and B (Table 1). MAb 4A8 recognized epitope A, whereas 4E2 and 2D5 recognized epitope B.

**Table 1:** Competitive binding of MAbs for the VP3 protein

**Competitor**	**HRP-labeled MAbs**		
	**4A8**	**4E2**	**2D5**
Epitope A			
4A8	+ +	––	––
Epitope B			
4E2	––	+ +	+ +
2D5	––	+ +	+ +

### Detection of VP3 antigens

MAbs 4A8 and 2D5, which recognized epitopes A and B, respectively were selected to test their cross-reactivity with other heterologous GPV strains (G3, GD, and HE) or MDPV (J3D6 and KL) in the ELISA. The relative binding to heterologous GPV or MDPV isolates was expressed as a percentage of the absorbance obtained with GPV EP22, which was set at 100. Binding was rated as strong if it was more than 50%, significant if it was 25%–50%, and negative if it was less than 25%. The results indicated that the VP3 in the cell extracts prepared from CGBQ cells infected with heterologous GPV strains or from MDEF cell infected with DPV was captured by the anti-VP3 antiserum. The binding rated for GPV and MDPV were 100% and there were no appreciable differences for GPV in the binding of the different MAbs tested. As expected, negative results were obtained with mock-infected CGBQ cells or DEF cells (< 25%). MAbs 4A8 and 2D5 strongly recognized all tested GPV and MDPV strains, suggesting that epitopes A and B are commonly present on the VP3 of GPV and MDPV strains and indicating that 4A8 and 2D5 are suitable for detecting GPV and MDPV isolates.

## Discussion

Here, we developed and characterized three MAbs against goose parvovirus VP3 protein. Our results show that antigen preparations containing the expressed His-VP3 protein of GPV could induce the production of MAbs. After screening and sub-cloning, three MAbs against His-VP3 were isolated and characterized. Western blotting and immunofluorescence assay indicated that the MAbs bound to the authentic viral VP3 protein of GPV and MDPV and that this protein localized mainly in the nucleus of infected cells, which is consistent with a previous report [22,23]. The MAbs bound to the His-VP3 in its native conformation, and when SDS and 2-mercaptoethanol were used to denature the His-VP3 protein, this binding was retained, indicating that the epitopes were not affected by breaking of disulfide bonds. This finding suggests that the MAbs binding was conformation-independent. Competitive binding assays were used to determine the epitopes recognized by the MAbs based on the notion that a MAb binding to a specific site can block the attachment of another MAb to the same site. Two epitopes, A and B, almost completely inhibited the binding of HRP-coupled MAbs recognizing the same epitope, but no competition was obtained among MAbs recognizing epitope A or B. Dot blotting analysis showed that the epitopes on the VP3 recognized by three MAbs were also present on the native viral VP3 of GPV and DPV particles, which is consistent with immunofluorescence assay analysis. By the immunofluorescence assay, it could also identify the GPV/MGPV antigen with MAbs because of its distinctive nuclear localization within infected cells, which consistent with previous report [22]. A similar finding was obtained when MAbs specificities were analyzed by Western blotting. Three major proteins (VP1, VP2 and VP3) MW are in consistent with previously reports of Muscovy duck (91, 78 and 58 kDa) and goose (85, 61 and 57 kDa) parvovirus virions [22-24].

Whether epitope A or B is involved in any biological function has not yet been determined; however, these epitopes, are highly conserved among GPV and MDPV strains, as the MAbs recognized both epitopes on all field strains tested. It has been suggested that the epitopes recognized by B19 [25] are involved in heparan sulfate binding. The region containing the epitope might function as the primary receptor attachment site of B19, yet it does not correspond to the globoside binding site [26]. This reason for this discrepancy requires clarification. Accordingly, to determine whether VP3 of GPV/MDPV is involved in heparan sulfate binding, we are currently preparing deletion mutant proteins of GPV/MDPV VP3 to assess with the MAbs described here.

Three MAbs successfully detected the native-form of the VP3 protein in infected cells, as well as in viral particles. Therefore, these MAbs may be useful in the development of sensitive methods to detect GPV and MDPV, such as immunoblot assays, immunofluorescence assays, and antigen-capture ELISA. Antigen-capture ELISA using antivirus antibodies is ideal for large screening, quantitative analysis of viral antigens or virus titres because of its high sensitivity, reproducibility, and automation. In this study, we generated three positive clones that secreted specific and highly reactive antibodies against VP3 protein for use in diagnostic methods. MAb-capture ELISA clearly differentiated between GPV-/MDPV- and mock-infected samples, as demonstrated by absorbance values, suggesting that non-specific reactions could be markedly reduced in a MAb-capture ELISA. Two of the MAbs (4A8 and 2D5) recognized GPV and MDPV VP3 at different sites that were highly conserved in all GPV and MDPV strains tested. Thus, a MAb-capture ELISA using MAbs 4A8 and 2D5 would appear to be an acceptable future screening method for the detection of GPV and MDPV in infected birds.

## Conclusion

In summary, the results of this study provide important information about MAbs against the GPV VP3 protein. In particular, the MAbs could contribute to the development of a MAb-capture ELISA for rapid detection of GPV and MDPV. GPV and DPV antigen could be detected with these MAbs in GPV infected cells in Immunofluorescence assay. Although virus isolation and PCR are reliable methods to detect GPV and MDPV infection, they are laborious, time-consuming, and requiring specialized instruments. These diagnosis problems highlight the need for alternative rapid, reproducible, and automatic methods to detect GPV and MDPV.

## Methods

### Cells and viruses

Heterologous goose parvoviruses EP22, G3, GD, HE, and Muscovy duck parvovirus (MDPV) J3D6 were used in this study. The GPV Ep22, G3, GD, and HE strains were isolated from the livers dead geese with hepatitis in China in 2001, 1995, 1989, and 2007, respectively, as described previously [27]. MDPV J3D6 and KL strains were isolated from dead Muscovy ducks in 1999 and 2008. VP3 gene of EP22, G3, GD, and HE diverge 4.1 to 4.7% at amino acids level. VP3 of J3D6 and KL showed 85.9%-88.7% identities to those of GPV. GPV were propagated in goose CGBQ cells (AATC CCL-169) or goose embryo fibroblast cells (GEF) or in the allantoic sacs of 14-day-old embryonated goose eggs. MDPV J3D6 and KL was propagated in 13-day-old Muscovy duck embryonated eggs.

### Virus purification

The allantoic fluids containing EP22 and J3D6 were centrifuged at 20 000 × *g* for 15 min. The supernatants were layered onto 30% (w/w) sucrose solution and concentrated by ultracentrifugation (109 000 × *g*, 10 h, 4°C). The pellets were suspended in PBS, clarified at 5000 × *g* for 20 min and ultracentrifuged at 109 000 × *g* for 2.5 h. The purified viruses were stored at −20°C until used.

### Antigen preparation

The VP3 protein used for the production and characterization of MAbs was synthesized in *Escherichia coli* BL21 (DE3) as described previously [17]. The expressed His-VP3 and 6.7-kDa His tag proteins were purified by using a Ni-NTA kit (Qiagen, Valencia, CA). The 6.7-kDa His tag protein was used as a negative control during screening for specific antibodies to VP3 in an ELISA.

### Monoclonal antibodies production

BALB/C mice (Harbin Veterinary Experimental Central) were immunized intraperitoneally with 30 μg of antigen containing the VP3 fusion protein in complete Freund’s adjuvant and boosted twice with the same amount of antigen in incomplete Freund’s adjuvant at 2-week intervals. Six weeks after the initial immunization and 4 days before the mice were sacrificed for the preparation of hybridomas, a final boost was given via the same route with 30 μg of the same antigen. MAbs were produced by using techniques similar to those described previously [28]. Briefly, spleens were removed from the immunized mice, and their splenocytes were fused with NS1 myeloma cells. Hybridoma cell lines secreting antibodies against the VP3 protein were screened and subcloned at least three times by use of a limiting dilution method and ascitic fluids were prepared with the cloned hybridomas in BALB/C mice. All mice were maintained in the animal facility at Harbin Veterinary Research Institute under standard conditions prescribed by the Institutional Guidelines. The study protocol was approved by the Institutional Animal Care and Use Committee.

### Serological screening

Hybridoma culture supernatants or mouse ascetic fluids were screened for antibodies in an indirect ELISA as described for the antibody binding assay. Antibodies that bound to the VP3 protein but failed to bind the 6.7-kDa protein were selected for sub-cloning.

### Isotyping

Isotypes of the produced MAbs were determined by using a Mouse Immunoglobulin isotyping kit (Zymed Laboratories, Inc.) according to the manufacturer’s instructions.

### Western blotting and dot blotting assays

The samples of expressed His-VP3/His proteins and purified GPV EP22 and MDPV J3D6 were denatured by boiling in SDS and 2-mercaptoethanol. The boiled samples were subjected to 10% SDS-PAGE and transferred to nitrocellulose membranes for Western blotting analysis. The membranes were probed with different MAbs followed by a secondary HRP-conjugated goat anti-mouse antibody (KPL, MD, USA). The purified GPV and MDPV antigen or blank allantoic fluids (as a negative control) were used for Western blotting assays. The native antigens containing His-VP3 and the 6.7 His protein (as a negative control) were used for dot blotting assays. The membranes were then probed with the same MAbs as for Western blotting assays.

### Detection of native VP3 protein by immunofluorescence assay

GEF and DEF infected with Ep22 and J3D6 strain (at 10 M.O.I. TCID50/cell), respectively, incubated at 37°C for 48 h. The cells were fixed with cold methanol for 10 min and then probed with different anit-VP3 MAbs and negative normal mouse serum for 1 h at 37°C. Bound antibodies were visualized using fluorescent conjugated antibodies against mouse IgG (1:500 dilutions) under a fluorescence microscope.

### Effect of native structure of VP3 on MAbs recognition

To determine if a native GPV or MDPV particles could be recognized by three MAbs, the purified GPV EP22 and MDPV J3D6 particles (about 1 μg) and blank allantoic fluids as negative control were spotted onto nitrocellulose membranes for dot blotting assays.

### Coupling of horseradish peroxidase to MAbs

Immunoglobulin fractions were isolated from ascetic fluids by precipitation at 4°C with an equal volume of saturated ammonium sulfate (pH 7.0), and then purified by using an affinity column of protein G-agarose (Boehringer Mannheim). Antibodies were coupled to HRP by means of the periodate method [29] and stored at -20°C.

### Determination of MAbs titres

The titers of the MAbs were determined by using an ELISA. The purified His-VP3 protein (0.1 μg) was coated onto plate wells at 37°C for 2 h. The plates (Nunc MaxiSorp® flat-bottom 96 well plate) were then washed three times with washing buffer (0.01 M phosphate-buffered saline, pH 7.2, 0.05% Tween 20) and blocked with 100 μl of TNE buffer containing 2.5% bovine serum albumin. After washing, two-fold serial dilutions of 1 μg/ml uncoupled or HRP-coupled MAbs were added and incubated for 1 h. For uncoupled MAbs, an additional 50 μl of HRP-coupled goat anti-mouse antibodies (KPL, MD, USA) was added. Absorbance was read at 405 nm with a Microplate Reader (BIO-RAD). The level of binding for the relative activity assessment was measured from the resulting dose–response curve.

### Antibody binding assay

For the competitive binding assay, the amount of MAb binding in the ELISA was determined for MAbs uncoupled or coupled with HRP [30]. Briefly, for HRP-unconjugated MAb determination, ELISA plates were coated with 0.1 μg of purified VP3 per well at 37°C for 2 h. After washing, 100 μl of TNE buffer containing 2.5% bovine serum albumin was added to each well to saturate all unbound sites. After washing, 100 μl of purified MAb serially diluted with TNE buffer containing 1% bovine serum albumin was added and incubated for 2 h at 30°C. After washing, 50 μl of a 1:500 dilution of HRP-conjugated goat anti-mouse IgG serum was added and incubated for another 1 h. The enzymatic activity was determined after 20 min of incubation by the addition of 30 ml of 1% sodium azide. Absorbance was measured at 405 nm. For HRP-conjugated MAb determination, the same procedures were carried out except that HRP-conjugated MAbs were directly added to the antigen-coated plates without using the HRP-conjugated goat anti-mouse antiserum. The level of maximum binding for the relative activity assessment and the MAb concentration at which 50% binding occurred were obtained from the resulting dose–response curve.

### Competitive binding assay

The competitive binding assay was performed similarly to the procedures described above, except that a mixture of the HRP-conjugated MAbs was used at twice the concentration, giving half-maximal binding. Unconjugated, competing antibodies at different concentrations were also added simultaneously. The competition between two MAbs for the same site was correlated to their relative avidities and concentrations. A spectrum of dose-related interference was tested. Non-specific binding without antigens was used to represent the background. The degree of competitive binding was measured from the absorbance at 405 nm in the presence or absence of unconjugated competing antibodies. Competition was rated as strong (++) if it was more than 60%, significant (+) if it was more than 30%, and negative (--) if it was less than 30%.

### Cross-reactivity of MAbs to heterologous GPV strains

To study the cross-reactivity of the MAbs for various GPV and MDPV strains in an antigen-captured ELISA, we tested three GPV (G3, GD, and HE) and MDPV (J3D6 and KL) field isolates. MAbs (4A8 and 2D5) were used to prepare an antigen-capture ELISA and compared with a polyclonal antibody against GPV EP22. Briefly, 100 μl of mouse anti-VP3 polyclonal antibodies (1:200) was coated onto ELISA plates. After washing and blocking, 100 μl of cell extracts of GEF or DEF infected with GPV or MDPV isolates or from mock-infected cells was added and incubated for 1 h at 37°C. For the MAb reactions, 50 μl of HRP-conjugated MAbs (1:1000) was added as the primary antibody. To determine whether the VP3 present in each cell extract from cells infected with each GPV and MDPV isolate was captured by the anti-VP3 antiserum, goose antiserum against GPV EP22 and HRP-coupled goat anti-goose antiserum were used as a primary and secondary antibodies, respectively. Absorbance was measured at 405 nm. Binding to the heterologous virus was expressed as a percentage of the absorbance obtained with GPV EP22, which was set at 100. Binding was rated as strong if it was more than 50%, significant if it was 25%–50%, and negative if it was less than 25%.

## Abbreviations

GPV: Goose parvovirus; MDPV: Muscovy duck parvovirus; MAb: Monoclonal antibody; ELISA: Enzyme-linked immunosorbent assay; PCR: Polymerase chain reaction.

## Competing interests

The authors declare that they have no competing interests.

## Authors’ contributions

ML and YZ are responsible for the research design and the writing of this manuscript. XCY, SMZ, JZL, SYT, HYL, XYW, and YHC performed the MAbs preparation and characterization, cloning, and sequencing of VP3 of the EP22 isolate. All authors read and approved the final manuscript. XCY and SMZ contribute equally to this paper.
